# Importance of remission and residual somatic symptoms in health-related quality of life among outpatients with major depressive disorder: a cross-sectional study

**DOI:** 10.1186/s12955-014-0188-y

**Published:** 2014-12-18

**Authors:** Jong-Min Woo, Hong Jin Jeon, Eunsun Noh, Hyo-Jin Kim, Sun Woo Lee, Kyung Kyu Lee, Sung Hwan Kim, Jin Pyo Hong

**Affiliations:** Department of Psychiatry, Seoul Paik Hospital, Inje University School of Medicine, Seoul, Korea; Stress Research Institute, Inje University, Seoul, Korea; Department of Psychiatry, Depression Center, Samsung Medical Center, Sungkyunkwan University School of Medicine, Seoul, Korea; Depression Clinical and Research Program, Massachusetts General Hospital, Harvard Medical School, Boston, USA; Veterans Affairs Medical Center, Providence, Rhode Island USA; Department of Pharmacy Practice, College of Pharmacy, University of Rhode Island, Kingston, Rhode Island USA; OR/RWD Team, Health and Value Division, Pfizer Pharmaceuticals Korea Limited, Seoul, Korea; Department of Psychiatry, Chungnam National University School of Medicine, Chungnam, Korea; Department of Psychiatry, Dankook University School of Medicine, Cheonan, Korea; Department of Psychiatry, Dong-A University School of Medicine, Busan, Korea; Department of Psychiatry, Asan Medical Center, Ulsan University College of Medicine, 388-1 Pungnap-2dong, Songpa-gu, Seoul 138-736 South Korea

**Keywords:** Major depressive disorder, Quality of life, EQ-5D, Q-LES-Q-SF

## Abstract

**Background:**

Major depressive disorder (MDD) is strongly associated with an impaired quality of life (QoL), which is itself affected by various factors. Symptom-oriented ratings poorly reflect the impact of disease on the QoL and level of functioning of the mental health of subjects. The purpose of this study was to assess health-related QoL (HRQoL) using preference-based measures in outpatients with MDD with regard to their remission achievement and clinical factors affecting the HRQoL.

**Methods:**

This was a cross-sectional observational study. We recruited 811 patients with MDD from 14 psychiatric outpatient clinics in Korea. They were divided into three groups as follows: a new visit group (n = 287), a remitted group (n = 235), and a non-remitted group (n = 289). The 17-item Hamilton Depression Rating Scale was used to assign patients to the remitted or non-remitted group. The general HRQoL was assessed with the EuroQol 5D (EQ-5D), using both the EQ-5D index score and the EuroQol Visual Analog Scale (EQ-VAS). The disease-specific HRQoL was assessed with the Quality of Life Enjoyment and Satisfaction Questionnaire Short Form (Q-LES-Q-SF).

**Results:**

The non-remitted group showed a significant impairment of HRQoL in view of the subscales of EQ-5D index scores, EQ-VAS, and Q-LES-Q-SF. The EQ-5D index score in the remitted group was 0.77 ± 0.10, while it was 0.57 ± 0.23 in the non-remitted group and 0.58 ± 0.24 in the new visit group (p < 0.0001). The EQ-VAS scores for the remitted and non-remitted groups were 72.5 ± 16.6 and 50.9 ± 20.3, respectively (p < 0.0001). Likewise, patients with remission had the Q-LES-Q-SF total score of 46.5 ± 8.8, whereas those with non-remission reported 36.7 ± 7.7 (p < 0.0001). The symptom severity measured by the Depression and Somatic Symptoms Scale was significantly correlated with the HRQoL. Furthermore, patients with severe somatic symptoms showed a significantly lower EQ-5D index score (0.54 ± 0.24) than those with mild/moderate somatic symptoms (0.75 ± 0.12; p = 0.002).

**Conclusion:**

Non-remitted MDD patients, especially those with more severe somatic symptoms, show a distinct impairment of HRQoL and more clinical symptoms, suggesting the importance of achieving remission in the treatment of MDD.

## Introduction

Major depressive disorder (MDD) is strongly associated with an impaired quality of life (QoL) and several studies have investigated the factors affecting this relationship [1-3].

Health related QoL (HRQoL) covers a wide range of dimensions including psychological status, functional abilities, subjective well-being, social interactions, role performance and physical health [4]. Due to this nature of HRQoL, assessment of HRQoL has received considerable research attention and provided potential for a more comprehensive evaluation of treatment outcomes.

However, symptom-oriented ratings poorly reflect the impact of mental health on QoL and level of functioning. Thus, QoL has been mainly assessed with patient-reported outcome (PRO) instruments. Recently, the importance of PRO measures in clinical trials for new drug, biologic agents and devices was underscored by the release of the US Food and Drug Administration's draft guidance for industry [5,6].

Recently, results of the Factors Influencing Depression Endpoints Research (FINDER) study of the European Union demonstrated that antidepressant treatment was associated with an improved HRQoL and that clinical factors, including the presence of somatic and painful symptoms, were negatively associated with HRQoL [1,7,8]. Depressed patients who fail to obtain full remission, which is defined as almost full recovery of depression symptoms to the level of a person without depression, show a higher risk of being depressed again and continue to experience functional impairment [9]. These results suggest that achieving remission and severity of certain residual symptoms can be more important for improving QoL.

Previous studies suggested an impaired HRQoL in patients with depression and an improved HRQoL in treated patients; however, the relationship between HRQoL, particularly measured using PRO and remission status is less well understood. In our current study, we quantified both generic and disease-specific measures of QoL and then compared them among three subgroups (new visit, remitted, and non-remitted groups). In addition, we examined the effect of HRQoL on suicidal thoughts and suicide attempts. We hypothesized that the non-remitted group would show more severe impairment in QoL than the new visit group and remitted group and, moreover, that the severity of somatic and pain symptoms would be closely associated with both generic and disease-specific QoL measures.

## Methods

### Subjects

The data of Korean patients with MDD were derived from the Korean Burden of Illness Study. This study was a non-interventional, multicenter-based, naturalistic observational, cross-sectional, and outpatient-based study of patients with MDD. All patients were enrolled from 14 regional psychiatric outpatient clinics in Korea. The study was conducted between December 2011 and September 2012. All study procedures were approved by the Institutional Review Board of Asan Medical Center and other sites. Written informed consent was obtained from all study patients before study initiation.

### Inclusion and exclusion criteria

Patients 18 years or older and meeting the DSM-IV criteria for a single or recurrent non-psychotic MDD were included. Each consecutive patient was evaluated according to the inclusion and exclusion criteria and was then allocated to one of three groups: (1) a new visit group, (2) a remitted group (17-item Hamilton Depression Rating Scale [HAM-D-17] <8), or (3) a non-remitted group (HAM-D-17 ≥ 8). Each hospital was allowed to consecutively allocate approximately eight patients to each group. Groups 2 and 3 included patients who had received MDD treatment for less than 6 months in order to exclude any bias possibility in calculating the lost productivity costs due to decreased social occupational function related to other emotional problems.

Exclusion criteria included history of bipolar disorder, schizophrenia, schizoaffective disorder, psychosis, anorexia or bulimia nervosa, obsessive-compulsive disorder, or a serious general medical condition.

### Measurement of depressive symptoms

The 17-item HAM-D was used to measure the severity of depressive symptoms at each visit [10]. The HAM-D-17 is a clinician-administered depression assessment scale, evaluating mood and vegetative symptoms [10]. Each item was rated with a score of 0-4 (where the scores are equivalent to absent, doubtful/trivial, mild, moderate and severe) or 0–2 (absent, doubtful/mild, and obvious, distinct/severe) [10]. By summing the score of each item, the total scores for HAM-D-17 can range from 0 to 54. In general, a total HAM-D-17 score of 7 or less is accepted as an indicator of remission [11]. It has been reported an internal consistency of 0.83 [12], inter-rater reliability of 0.97 [13], and test-retest reliability of 0.81 [14], showing adequate reliability.

### Measurement of somatic symptoms

The Depression and Somatic Symptoms Scale (DSSS) was applied to measure somatic symptoms in MDD patients [15]. The DSSS is a 22-item self-administered rating scale containing three subscales: a Depression Subscale (DS), a Pain Subscale (PS), and a Somatic Subscale (SS). The DS has 12 items, including three vegetative symptoms and fatigue, whereas the SS has 10 items, including five pain items composed of the 5-item PS. Each item was rated with a score of 0–3: 0 (not at all), 1 (mild), 2 (moderate), or 3 (severe). Thus, the range of summed scores was 0–66. The scale shows a good validity and reliability, with higher scores indicating stronger symptoms. The Korean Version of DSSS has a Cronbach's alpha of 0.90 and shows a relatively high test-retest reliability (r = 0.83, p < 0.01) [16,17].

### Measures of QoL

In order to assess HRQoL, we used generic instruments, including the European Quality of Life-5 Dimensions (EQ-5D; containing an index score and visual analog scale [VAS]), and disease- or condition-specific QoL measures, including the Quality of Life Enjoyment and Satisfaction Questionnaire (Q-LES-Q).

#### EuroQol (EQ-5D and EQ-VAS)

EQ-5D is a standardized preference-based measure of health status developed by the EuroQol Group that is applicable to mental health conditions such as mild to moderate depression and anxiety [18-20]. The EQ-5D was developed to provide a simple, generic measure of health for clinical and economic appraisals [20]. It provides a simple descriptive profile and a single index value for health status that can be used in the clinical and economic evaluation of health care as well as in population health surveys. The EQ-5D is designed for self-completion by respondents and essentially consists of two pages. The EQ-5D descriptive system comprises the following five dimensions (with three levels) and the EQ visual analog scale (EQ-VAS). The EQ-5D consists of five dimensions (mobility, self-care, usual activities, pain/discomfort, anxiety/depression), each with three levels of severity in health utility (no problems/some or moderate problems/extreme problems) recording an individual’s ratings of EQ-5D health states. The Korean version of the EQ-5D was developed and validated by Kim et al. in 2005 [21]. The EQ-5D index score was calculated based on the weights elicited from a large national survey of the Korean population [22].

#### The Q-LES-Q

The Q-LES-Q is a self-report instrument designed to measure satisfaction and enjoyment in various domains of functioning (e.g., physical health, work, and household duties) [23]. It can be applied to depressed individuals [24]. Many studies have used the Q-LES-Q to measure life satisfaction and enjoyment in patients with depression during pre- and post-treatment phases [23,25,26]. The Q-LES-Q Short Form (Q-LES-Q-SF) used in this study consists of 16 items, of which the first 14 assess discrete domains such as social relationships, living or housing situation, and physical health. Item 15 concerns the respondents’ satisfaction with medication they are taking, if applicable. Item 16 is a global rating in which respondents are asked to rate their “overall life satisfaction and contentment.” Each item is scored on a 5-point Likert scale that indicates the degree of enjoyment or satisfaction achieved during the past week (1 = very poor, 5 = very good). Higher scores indicate better function. Items 1 through 14 of the Q-LES-Q-SF can be summed to obtain a total score, with higher scores indicating greater life enjoyment and satisfaction. For the present analyses, we used the total score generated by the sum of the first 14 items. This scale has a Cronbach’s alpha of 0.90 and a test-retest reliability of 0.74 [23]. The construct validity of the Q-LES-Q is supported by moderately negative correlations with the Clinical Global Impressions–Severity of Illness scale (CGI-S) (r = –0.62 for the summary scale) and the 17-item HAM-D, compared in a depressed population [23]. To compare with other instruments of HRQoL we also obtained the Q-LES-Q index score by converting the Q-LES-Q total score to a percentage.

### Measures of suicidal thoughts and suicide attempts

We assessed suicide attempts and suicidal thoughts during the past month before study inclusion using the Korean version of the Mini-International Neuropsychiatric Interview (MINI) suicidality module [27]. The MINI suicidality module is composed of six questions including wish for death, wish for self-harm, suicidal thoughts, suicide plans, suicide attempts in the past month, and lifetime suicide attempts. We included the Mini-C2 (suicide attempts in the past month) and Mini-C3 (suicidal thoughts) in the analysis to examine the association between HRQoL and suicidal thoughts/suicide attempts.

### Statistical analysis

Descriptive statistics (mean, standard deviation) were obtained for continuous data in each MDD patient group. ANOVA (or Kruskal-Wallis test) was performed to compare the patient groups. Tukey’s multiple comparison method was used for post-hoc group comparisons after the significance for ANOVA was determined. The QoL was compared between the MDD patient groups using ANCOVA, controlling for the depression symptom score as covariate. The association between the QoL and depression symptoms score was evaluated by stepwise regression using the full set of variables of the DSSS adjusted by age and sex. Additionally, the association between suicidal thoughts/suicide attempts and HRQoL using logistic regression analysis was adjusted by age and DSSS score. Frequency and percentage were obtained for categorical data and the chi-square test was applied to the categorical data. All analyses were performed using SAS® version 9.2 (SAS Institute, Cary, NC).

## Results

### Baseline characteristic of the study subjects

A total of 811 patients with MDD were included in this study, 235 in the remitted group, 289 in the non-remitted group, and 287 in the new visit group. The demographics and clinical characteristics of study subjects are presented in Table 1. Approximately 70% of the patients were women in each group and the mean age ranged from 44.4 to 47.6 years across the groups. There were 62.3% unemployed subjects in the non-remitted group while the remitted group showed an unemployment rate of 57.4%. In addition, patients in the non-remitted group demonstrated a slightly higher number of depressive episodes than the patients in the remitted group (mean ± standard deviation [SD], 1.3 ± 2.2 vs. 1.7 ± 2.6) and a slightly longer duration of MDD (7.3 ± 16.6 vs. 8.1 ± 20.5 months).

**Table 1:** **Demographics of the study subjects in the new visit, remitted, and non-remitted MDD groups**

	**New MDD (N = 287)**	**Remitted MDD (N = 235)**	**Non-remitted MDD (N = 289)**	**p-value**	**Test** ^**a**^
Sex, n (%)					
Male	87 (30.3)	60 (25.5)	84 (29.1)	NS	
Female	200 (69.7)	175 (74.5)	205 (70.9)		
Age (years)	44.4 ± 13.6	47.6 ± 11.8	44.8 ± 13.3	0.0111^b^	a = c < b
Marital status, n (%)					
Single	63 (22.0)	36 (15.3)	62 (21.5)	NS	
Married	184 (64.1)	165 (70.2)	182 (63.0)		
Bereaved	11 (3.8)	12 (5.1)	21 (7.3)		
Divorced	24 (8.4)	15 (6.4)	18 (6.2)		
Separated	4 (1.4)	7 (3.0)	6 (2.1)		
Education, n (%)					
Elementary school	40 (13.9)	32 (13.6)	46 (15.9)	NS	
Middle school	51 (17.8)	31 (13.2)	38 (13.1)		
High school	107 (37.3)	105 (44.7)	121 (41.9)		
University	81 (28.2)	56 (23.8)	72 (24.9)		
Graduate school	7 (2.4)	11 (4.7)	12 (4.2)		
Job status, n (%)					
Employed	128 (44.6)	100 (42.6)	109 (37.7)	NS	
Unemployed	159 (55.4)	135 (57.4)	180 (62.3)		
Onset of MDD (years)	41.6 ± 14.2	44.7 ± 12.6	41.3 ± 14.2	0.0111^b^	a = c < b
Number of depressive episodes	1.1 ± 2.0	1.3 ± 2.2	1.7 ± 2.6	0.0131^b^	a = b, b = c, a < c
Duration of depression (months)	6.3 ± 13.7	7.3 ± 16.6	8.1 ± 20.4	NS	

MDD = major depressive disorder; NS = not significant.

^a^Tukey’s post-hoc test.

^b^Difference among MDD groups (ANOVA).

Note: Data are represented as mean ± standard deviation or number of patients (%).

### Disease severity of the subjects

Table 2 shows the symptom severity measured by 17-item HAM-D scores and DSSS. Patients who achieved remission showed a HAM-D score of 4.5 ± 2.1, which is significantly lower than the scores of the non-remitted group (16.6 ± 5.8) and the new visit group (17.8 ± 7.0) (p < 0.0001). Similarly, the total scores of depressive and somatic symptoms were significantly lower in the remitted group than in the non-remitted and new visit group. The depressive symptom scores in the remitted group were 5.9 ± 5.3, whereas they were 16.8 ± 7.2 in the non-remitted group and 17.4 ± 7.2 in the new visit group (p < 0.0001). In addition, the total score of somatic symptoms including pain was 4.0 ± 3.9 in the remitted group, 10.6 ± 6.2 in the non-remitted group, and 10.9 ± 5.8 in the new visitors (p < 0.0001).

**Table 2:** **Severity of depressive and somatic symptoms**

	**New MDD (N = 287)**	**Remitted MDD (N = 235)**	**Unremitted MDD (N = 289)**	**p-value**	**Test** ^**a**^
HAM-D scores	17.8 ± 7.0	4.5 ± 2.1	16.6 ± 5.8	<0.0001^b^	b < c < a
Bech scores^c^	8.1 ± 3.5	2.0 ± 1.6	7.5 ± 2.9	<0.0001^b^	b < c < a
Maier-Philipp scores^d^	8.2 ± 3.7	1.8 ± 1.5	7.4 ± 3.2	<0.0001^b^	b < c < a
Gibbons scores^e^	10.7 ± 4.6	2.5 ± 1.7	10.0 ± 4.1	<0.0001^b^	b < c = a
Suicidality scores	9.3 ± 8.3	6.9 ± 7.4	10.8 ± 8.8	0.0008^b^	a = b, a = c, b < c
DS score	17.4 ± 7.2	5.9 ± 5.3	16.8 ± 7.2	<0.0001^b^	a = c < b
SS score	10.9 ± 5.8	4.0 ± 3.9	10.6 ± 6.2	<0.0001^b^	a = c < b
PS score	5.2 ± 3.1	2.2 ± 2.2	5.1 ± 3.4	<0.0001^b^	a = c < b
DSSS total score	28.2 ± 11.8	9.9 ± 8.5	27.3 ± 12.3	<0.0001^b^	a = c < b

MDD = major depressive disorder; HAM-D = Hamilton depression rating scale; DSSS = depression and somatic symptoms scale; DS = depression subscale of the DSSS; SS = somatic subscale of the DSSS; PS = pain subscale of the DSSS.

^a^Tukey’s post-hoc test.

^b^Difference among MDD groups (ANOVA).

^c^Depressed mood, feeling of guilt, work and activities, retardation, anxiety/psychic, and general somatic symptoms are included.

^d^Depressed mood, feeling of guilt, work and activities, retardation, agitation, and anxiety/psychic are included.

^e^Depressed mood, feeling of guilt, suicide, work and activities, anxiety/psychic, agitation, anxiety (somatic), and genital symptoms are included.

Note: Data was represented as mean ± standard deviation.

### HRQoL

The HRQoL measured by the EQ-5D index and EQ-VAS and the disease-specific QoL measured by the Q-LES-Q-SF are described in Table 3. The EQ-5D index score was 0.77 ± 0.10 in the remitted group and 0.57 ± 0.23 in the non-remitted group (p < 0.0001). Pain/discomfort and anxiety/depression were the two strongest influencers of patients' HRQoL among each dimension of EQ-5D. Approximately 65% of patients in remission reported no problems with pain/discomfort, whereas more than 66% of patients in the non-remitted group reported some/severe problems with pain/discomfort. Similar to the pain/discomfort domain, approximately 61% of the remitted group reported no problems with anxiety/depression, whereas 87.2% of the non-remitted group documented some/severe problems. The HRQoL measured by EQ-VAS was comparable to the EQ-5D index score in each group; it was 72.5 ± 16.6 in the remitted group and 50.9 ± 20.3 in the non-remitted group (p < 0.0001). Life satisfaction measured by Q-LES-Q-SF was approximately 10 points higher in the remitted group than in the non-remitted group (46.5 ± 8.8 vs. 36.7 ± 7.7; p < 0.0001). More than 95% of MDD patients were treated with antidepressant medication and 70.8% of the remitted group were satisfied/very satisfied with their antidepressant medication therapy. However, only 41.8% of non-remitted patients reported that they were satisfied/very satisfied with their medication.

**Table 3:** **HRQoL (EQ-5D, EQ-VAS, Q-LES-Q-SF)**

	**New MDD (N = 287)**	**Remitted MDD (N = 235)**	**Non-remitted MDD (N = 289)**	**p-value**	**Test** ^**a**^
EQ-5D dimensions mobility, n (%)					
No problems	246 (85.7)	219 (93.2)	237 (82.0)		
Some problems	35 (12.2)	16 (6.8)	49 (17.0)		
Severe problems	6 (2.1)	0 (0.0)	3 (1.0)		
Self-care, n (%)					
No problems	268 (93.4)	228 (97.0)	267 (92.4)		
Some problems	19 (6.6)	7 (3.0)	21 (7.3)		
Severe problems	0 (0.0)	0 (0.0)	1 (0.3)		
Usual activity, n (%)					
No problems	155 (54.0)	201 (85.5)	143 (49.5)		
Some problems	119 (41.5)	33 (14.0)	130 (45.0)		
Severe problems	13 (4.5)	1 (0.4)	16 (5.5)		
Pain/discomfort, n (%)					
No problems	98 (34.1)	153 (65.1)	96 (33.2)		
Some problems	163 (56.8)	79 (33.6)	152 (52.6)		
Severe problems	26 (9.1)	3 (1.3)	41 (14.2)		
Anxiety/depression, n (%)					
No problems	28 (9.8)	143 (60.9)	37 (12.8)		
Some problems	189 (65.9)	90 (38.3)	192 (66.4)		
Severe problems	70 (24.4)	2 (0.9)	60 (20.8)		
EQ-5D index score^c^	0.58 ± 0.24	0.77 ± 0.10	0.57 ± 0.23	<0.0001^b^	a = c < b
EQ-VAS	50.3 ± 20.6	72.5 ± 16.6	50.9 ± 20.3	<0.0001^b^	a = c < b
Q-LES-Q-SF total score^d^	35.6 ± 7.8	46.5 ± 8.8	36.7 ± 7.7	<0.0001^b^	a = c < b
Q-LES-Q-SF index score^e^	0.39 ± 0.14	0.58 ± 0.16	0.41 ± 0.14	<0.0001^b^	a = c < b
Status of concomitant medication, n (%)					
Yes	96 (33.4)	226 (96.2)	280 (96.9)		
No	191 (66.6)	9 (3.8)	9 (3.1)		
Satisfaction with medication, n (%)					
Very dissatisfied	2 (2.1)	0 (0.0)	5 (1.8)		
Dissatisfied	33 (34.4)	14 (6.2)	52 (18.6)		
Fair	32 (33.3)	52 (23.0)	106 (37.9)		
Satisfied	27 (28.1)	136 (60.2)	106 (37.9)		
Very satisfied	2 (2.1)	24 (10.6)	11 (3.9)		
Overall life satisfaction and contentment, n (%)					
Very poor	42 (14.6)	1 (0.4)	27 (9.3)		
Poor	141 (49.1)	25 (10.6)	120 (41.5)		
Fair	87 (30.3)	97 (41.3)	111 (38.4)		
Good	16 (5.6)	90 (38.3)	28 (9.7)		
Very good	1 (0.3)	22 (9.4)	3 (1.0)		
Overall life satisfaction and contentment	2.3 ± 0.8	3.5 ± 0.8	2.5 ± 0.8	<0.0001^b^	a < c < b

HRQoL = health-related quality of life; MDD = major depressive disorder; EQ-5D = European quality of life-5 dimensions; EQ-VAS = European quality of life visual analog scale; Q–LES-Q-SF = the quality of life enjoyment and satisfaction questionnaire short form.

^a^Tukey’s post-hoc test.

^b^Difference among MDD groups (ANOVA).

^c^EQ-5D index score = 1(0.164 + 0.003 × M2 + 0.274 × M3 + 0.058 × SC2 + 0.078 × SC3 + 0.045 × UA2 + 0.134 × UA3 +0.049 × PD2 + 0.132 × PD3 + 0.044 × AD2 + 0.102 × AD3 + 0.345 × N3 + 0.014 × I2sq).

Where, M2 = dummy for mobility level 2, M3 = dummy for mobility level 3, SC2 = dummy for self-care level 2, SC3 = dummy for self-care level 3, UA2 = dummy for usual activity level 2, UA3 = dummy for usual activity level 3, PD2 = dummy for pain/discomfort level 2, PD3 = dummy for pain/discomfort level 3, AD2 = dummy for anxiety/depression level 2, AD3 = dummy for anxiety/depression level 3, N3 = 1 if there is any level 3, otherwise N3 = 0, I2sq = the square of 1 minus the number of level 2s.

^d^Summing only the first 14 items to yield a raw total score.

Each item is calculated as 1 = very poor, 2 = poor, 3 = fair, 4 = good, 5 = very good.

^e^Q-LES-Q-SF index score = (Q-LES-Q-SF total score – 14)/56.

Note: Data are represented as mean ± standard deviation or number of patients (%).

### Relationship between depression subscales and HRQoL

The results of multiple stepwise regression analysis between the HRQoL and depression subscales are demonstrated in Table 4. When adjusted for age and sex, the symptom severity measured by the DSSS was significantly associated with the HRQoL. The EQ-5D index score decreased by 0.004 in the remitted group (p = 0.0340) and 0.017 in the non-remitted group (p < 0.0001) for every unit increase in the somatic symptom score.

**Table 4:** **Results of stepwise multiple regression analysis: Association between depression and somatic symptoms subscales and the quality of life**

**Variable**	**New MDD (N = 287)**		**Remitted MDD (N = 235)**		**Non-remitted MDD (N = 289)**		**Total (N = 811)**	
	**Coefficient**	**p-value**	**Coefficient**	**p-value**	**Coefficient**	**p-value**	**Coefficient**	**p-value**
DS score	−0.012	<0.0001	−0.010	<0.0001	-	-	−0.008	<0.0001
SS score	−0.006	0.0173	−0.004	0.0340	−0.016	<0.0001	−0.009	<0.0001
Bech score	−0.012	0.0012	-	-	-	-	-	-
Gibbons score	-	-	-	-	−0.017	<0.0001	−0.008	<0.0001

MDD = major depressive disorder; DS = depression subscale of the depression and somatic symptoms scale (DSSS); SS = somatic subscale of the DSSS.

Stepwise regression analysis using the full set of variables of the DSSS adjusted by age and sex:

Model 1: QoL (EQ-5D index score|New) = a + b1(DS score) + b2(SS score) + b3(Bech) + b4(Age) + b5(Sex).

Model 2: QoL (EQ-5D index score|Remitted) = a + b1(DS score) + b2(SS score) + b3(Age) + b4(Sex).

Model 3: QoL (EQ-5D index score|Non-remitted) = a + b1(SS score) + b2(Gibbons) + b3(Age) + b4(Sex).

Table 5 presents the HRQoL by the level of somatic symptoms in patients over 40 years of age. Depressed adults with severe somatic symptoms (≥8) reported a significantly lower EQ-5D index score than those with mild/moderate somatic symptoms (<8). This is shown through the severity of their somatic symptoms: 0.54 ± 0.24 in those with severe symptoms versus 0.75 ± 0.12 in those with mild/moderate symptoms (p = 0.0017), if adjusted for depression symptoms. Specifically, patients with mild/moderate somatic symptoms showed an EQ-5D index score of 0.72 ± 0.14 in the non-remitted group, close to the average EQ-5D index score in the remitted group. Regarding disease-specific HRQoL, patients with severe somatic symptoms presented an approximately 20% lower mean difference in the Q-LES-Q-SF index score than those with mild/moderate somatic symptoms (0.38 ± 0.14 vs. 0.48 ± 0.13).

**Table 5:** **Quality of life by somatic symptom severity**

**Variable**	**Overall** ^**a**^			**Remitted MDD**			**Non-remitted MDD**		
	**SS ≥ 8 (n = 277)**	**SS < 8 (n = 279)**	**p-value** ^**b**^	**SS ≥ 8 (n = 28)**	**SS < 8 (n = 155)**	**p-value** ^**b**^	**SS ≥ 8 (n = 118)**	**SS < 8 (n = 67)**	**p-value** ^**b**^
EQ-5D index score	0.54 ± 0.24	0.75 ± 0.12	0.0017*	0.65 ± 0.19	0.79 ± 0.07	0.0139*	0.52 ± 0.24	0.72 ± 0.14	0.0015*
EQ-VAS	49.32 ± 20.57	69.03 ± 17.56	0.0003*	61.29 ± 15.69	75.50 ± 16.18	0.0372*	47.79 ± 20.68	59.67 ± 16.91	0.2218
Q–LES-Q-SF index score	0.38 ± 0.14	0.55 ± 0.15	0.0008*	0.48 ± 0.13	0.60 ± 0.16	0.9300	0.38 ± 0.14	0.49 ± 0.12	0.2973

MDD = major depressive disorder; SS = somatic subscale of the depression and somatic symptoms scale; EQ-5D = European quality of life-5 dimensions; EQ-VAS = European quality of life visual analog scale; Q–LES-Q-SF = the quality of life enjoyment and satisfaction questionnaire short form.

^a^Patients aged 40 years or older.

^b^The difference between groups was derived from ANCOVA adjusted for depression symptom scores.

^*^Significance level p < 0.05.

### Association between HRQoL and suicidal thoughts/suicide attempts

Depression severity and impaired HRQoL were significantly associated with an increased risk of suicidal thoughts and suicide attempts (Table 6). Non-remitted patients showed a significantly higher risk of having suicidal thoughts during the past month than those in remission (odds ratio [OR] 2.55; 95% confidence interval [CI], 1.50–4.33; p = 0.0006). Similarly, non-remitted patients showed a significantly higher risk of having made a suicide attempt during the past month than those in remission (OR 2.14; 95% CI, 1.08–4.25; p = 0.0289). Furthermore, a one-unit improvement in the EQ-5D index score had a 70% odds reduction of suicidal thoughts (OR 0.3; 95% CI 0.12–0.75; p = 0.0104) and a 79% odds reduction of suicide attempts (OR 0.21; 95% CI 0.08–0.57; p = 0.0023) in depressed patients.

**Table 6:** **Association between suicidal thoughts/suicide attempts and disease severity and HRQoL**

**Variable**	**Suicidal thoughts**	**Suicide attempts**
	**OR** ^**a**^ **(95% CI)**	**OR** ^**a**^ **(95% CI)**
Groups by depression severity		
Remitted	Reference	Reference
Non-remitted	2.55 (1.50–4.33)	2.14 (1.08–4.25)
New visit	1.56 (0.90–2.70)	1.21 (0.59–2.47)
EQ-5D index score	0.30 (0.12–0.75)	0.21 (0.08–0.57)

EQ-5D = European quality of life-5 dimensions; OR = odds ratio; CI = confidence interval.

^a^Adjusted for age and DSSS score.

## Discussion

### Overall results

Here, we examined the HRQoL by the remission status of depressed patients in Korea. In the present study, the non-remitted group showed a significant impairment in HRQoL in view of the subscales of EQ-5D index scores and the EQ-VAS. Similarly, the life satisfaction measured by the Q-LES-Q-SF was significantly lower in the non-remitted group than in the remitted group. Even though patients in both groups were treated for their current symptoms of depression, patients who did not reach the treatment goals showed a significantly worse HRQoL than those who met the goals. This finding may suggest that remission achievement in depression plays an important role in the improvement in the preference-based outcomes of patients.

### Impact of disease severity on HRQoL

Previous research in France assessing the HRQoL using the EQ-5D in primary care settings reported EQ-5D index scores of 0.85 for patients in remission, 0.72 for non-remitted patients, and 0.58 for patients who had not responded to the treatment at the 8-week follow-up [28]. Moreover, the FINDER study documenting the impact of antidepressant medication treatment on patients' HRQoL found mean EQ-5D index scores of 0.75 at the 6-month follow-up [8]. Several other studies evaluating HRQoL in depressed patients using various instruments also presented similar utility scores when patients achieved remission: 0.79 measured by McSad [29], 0.70 by quality of well-being [30], and 0.74 by the standard gamble method [31].

In our study, the generic HRQoL measured via the EQ-5D in the remitted group was consistent with the previous research; however, the HRQoL in the non-remitted group was lower than that of previous studies. The EQ-5D index score in our present study was similar to the estimated EQ-5D value of the treatment non-respondent group in the study conducted by Sapin et al. [28]. There is evidence suggesting that having depression impairs the HRQoL [32] and that antidepressant medication therapy helps to improve HRQoL in depressed patients by decreasing MDD symptoms [8,33]. In our current study, whilst more than 95% of patients in both the remitted and non-remitted groups were treated with antidepressant medication, satisfaction toward their medication was lowered in the non-remitted group: one of every five patients in the non-remitted group was dissatisfied with their medication use and only 42% expressed satisfaction in this group. Thus, most patients in the non-remitted group may not respond to their therapy and, therefore, their HRQoL is comparable to the value reported for non-responders.

### Impact of somatic symptoms on HRQoL

Residual somatic symptoms could be another possible explanation for the worse HRQoL in the non-remitted group compared with those of other studies. In our subset analyses, after controlling for depression symptom scores, patients with severe somatic symptoms had significantly worse HRQoL than those who had mild/moderate somatic symptoms. Even non-remitted patients with mild/moderate somatic symptoms showed comparable EQ-5D index scores to those in remission. This finding indicates that residual somatic symptoms highly influence our patient-reported outcomes.

Several investigators have reported that the presence of somatic symptoms, including painful physical symptoms (PPS), is associated with greater depression severity and poor HRQoL [33-35]. Fava et al. [33] examined the effect of antidepressant medication on PPS in depressed patients, demonstrating that improvements in the PPS were linked to higher remission rates. Other studies showing the association between PPS and HRQoL suggested that patients with positive PPS at the baseline presented 10%–15% lower HRQoL scores (EQ-VAS of 42.2–43.0) than those with negative PPS (EQ-VAS of 52.9–60.4) [34,35]. The presence of PPS is still associated with less improvement in HRQoL when adjusted for depression severity [1,36].

Co-existing pain in depressed patients is highly prevalent and more likely to be underdiagnosed and undertreated because of the presence of the depression [37]. The prevalence of co-existing PPS in depressed patients ranges from 43% to 73%, varying by country [35,38-40]. In particular, 52% of patients with depression in Asia reported having positive PPS [35]. Despite this awareness, more than 80% of patients suffering from PPS were not prescribed concomitant treatment for their pain management [35]. Interestingly, the health condition with the largest negative impact on the HRQoL in the general population is pain, followed by depression, osteoarthritis, and anxiety [32]. Due to depressive symptoms or co-existing somatic symptoms, including PPS, depressed patients may be at higher risk of an impaired HRQoL. Therefore, physicians or other health care providers working with depressed patients should pay careful attention to somatic symptoms as well as classical depressive symptoms if such patients are seeking help regarding their depression.

### Comparison between preference-based and disease-specific HRQoL

In our present study, subjects showed lower Q-LES-Q-SF scores as disease-specific QoL measures than those of previous studies. The scores were 46.5% in the remitted group and 36.7% in the non-remitted group, whereas previous studies reported Q-LES-Q-SF scores of 52%–54% in patients with depression and 74%–79% in subjects that had once had depression [41,42]. The discrepancy may be related to the different levels of severity between the studies. Previous investigations assessed the disease-specific QoL in patients with chronic major depression or dysthymia, whereas it was assessed in subjects with more severe depression in university hospital settings in our study.

### HRQoL and suicidal thoughts/suicide attempts

In general, QoL is measured on a scale from 0 (deceased) to 1 (completely healthy), indicating that death is the worst state that people can imagine [19]. An impaired HRQoL is also significantly associated with both suicidal thoughts and suicide attempts [43]. One study using a population-based data survey showed that people with an EQ-5D index score less than 0.7 were 3.4 times more likely to attempt suicide than those with an index score of 0.8–1.0 [43]. This study also reported that people with an EQ-5D index score less than 0.7 had a 9.1 times increased risk of suicidal thoughts than those with a score between 0.8–1.0. Our study found a comparable pattern of association in patients with depression. Non-remitted patients with a worse HRQoL showed a more than 2-fold increased risk of suicidal thoughts and suicide attempts than those in remission. Furthermore, the improvement in EQ-5D index scores significantly reduced both suicidal thoughts and suicide attempts in depressed patients. Our findings indicate the importance of achieving remission in the treatment of depressed patients, not only for the improvement of HRQoL, but also for the prevention of the worst outcomes, such as suicide or death.

### Strengths and limitations of the study

Our study has several limitations. First, due to the nature of a cross-sectional study, we were unable to address the improvement in HRQoL according to the type of treatment or total duration. However, our investigation involved multiple sites in Korea, reflecting a similar cohort to a real world population with depression. Furthermore, by recruiting the new visit group, we attempted to show the initial baseline characteristics of patients who visited the hospitals. Second, the depression treatment was provided based on the health care delivery system of each site. The possible differences between sites might have affected our HRQoL results. To minimize unintended bias, we recruited the same number of patients from each site.

## Conclusions

We evaluated the HRQoL by disease severity in patients with depression. Non-remitted MDD patients with more severe somatic symptoms suffer from a poorer HRQoL and have more clinical symptoms, emphasizing the importance of achieving remission in the treatment of subjects with MDD in order to improve their HRQoL. In addition, the presence of somatic symptoms is significantly associated with impairment of HRQoL, suggesting that somatic symptoms of patients should be carefully evaluated to improve the preference-based QoL.

## Abbreviations

DS: Depression subscale of the depression and somatic symptoms scale
DSSS: The depression and somatic symptoms scale
EQ-5D: European quality of life-5 dimensions
EQ-VAS: European quality of life visual analog scale
HAM-D: Hamilton depression rating scale
HAM-D-17: 17 items Hamilton depression rating scale
HRQoL: Health-related quality of life
MDD: Major depressive disorder
MINI: The mini-international neuropsychiatric interview
PS: Pain subscale of the depression and somatic symptoms scale
Q–LES-Q: The quality of life enjoyment and satisfaction questionnaire
Q–LES-Q-SF: the quality of life enjoyment and satisfaction questionnaire short form
QoL: Quality of life
SS: Somatic subscale of the depression and somatic symptoms scale
